# Book Notices

**Published:** 1884-03

**Authors:** 


### BOOK NOTICES.

### Letters from a Mother to a Mother on the Formation, Growth, and Care of the Teeth, by the Wife of a Dentist, Mrs. M. W. J.

These letters—sixteen in number—have been, except the last three, published in the *Southern Dental Journal*. They are now published in a pamphlet of seventy-two pages. Scarcely anywhere have we seen so much practical, useful matter in so condensed form—useful to the profession—but especially adapted to the class of persons for whom it was written.

It is eminently adapted for distribution by the dentist to his patients, particularly to those who are, or are to be, mothers. A better service can hardly be done than to distribute such information as is contained in this work.

It is published by, and can be obtained of, Messrs. Judson & Dunlop, Atlanta, Ga.

Price, $1.50 per dozen.

---

The transactions of the twenty-third annual meeting of the American Dental Association, issued by The S. S. White Manufacturing Co., is just at hand.

It is a volume of 118 pages, well gotten up, and the printing and paper excellent.

The proceedings and papers are given in full, and also a very faithful report of the discussions. Every member of the profession who desires to keep up with the progress of the times should have every such volume in his library, and especially the transactions of the American Dental Association.

The thanks of the members of the Association are due to the Publication Committee for the manner in which they have performed their work, and especially to The S. S. White Manufacturing Co., who have executed the work so neatly, and without cost to the Association.

It can be obtained of Dr. George W. Cushing, Chicago, or of The S. S. White Manufacturing Co., Philadelphia.

---

THE transactions of the last annual meeting of the Ohio State Dental Association is just from the press, giving the proceedings, papers, and discussions; of the latter two, there was much less than usual at this meeting. More time was consumed by routine and special business than has been common. The special business was of more than ordinary importance, as will be seen by looking over these transactions. Every member of the profession in the State should be familiar with the work of the last meeting of this Society.

Copies may be obtained by addressing Dr. J. H. Warner, Columbus, Ohio.

